# Dispensed prescription medications and short-term risk of pulmonary embolism in Norway and Sweden

**DOI:** 10.1038/s41598-024-69637-4

**Published:** 2024-08-29

**Authors:** Dagfinn Aune, Ioannis Vardaxis, Bo Henry Lindqvist, Ben Michael Brumpton, Linn Beate Strand, Jens Wilhelm Horn, Inger Johanne Bakken, Pål Richard Romundstad, Kenneth J. Mukamal, Rickard Ljung, Imre Janszky, Abhijit Sen

**Affiliations:** 1 Department of Epidemiology and Biostatistics, School of Public Health, Imperial College London, St. Mary’s Campus, Norfolk Place, Paddington, London, W2 1PG UK; 2 Department of Nutrition, Oslo New University College, Oslo, Norway; 3 Department of Research, Cancer Registry of Norway, Oslo, Norway; 4 Department of Mathematical Sciences, Norwegian University of Science and Technology, Trondheim, Norway; 5 Department of Thoracic Medicine, St. Olav’s Hospital, Trondheim University Hospital, Trondheim, Norway; 6 K.G. Jebsen Centre for Genetic Epidemiology, Department of Public Health and Nursing, Norwegian University of Science and Technology, Trondheim, Norway; 7 MRC Integrative Epidemiology Unit, University of Bristol, Bristol, UK; 8 Department of Public Health and Nursing, Faculty of Medicine and Health Sciences, Norwegian University of Science and Technology, 7491 Trondheim, Norway; 9 Department of Internal Medicine, Levanger Hospital, Health Trust Nord-Trøndelag, Levanger, Norway; 10 Department of Health Registries, Norwegian Directorate of Health, Trondheim, Norway; 11 Department of Medicine, Beth Israel Deaconess Medical Center, Boston, MA USA; 12 Unit of Epidemiology, Institute of Environmental Medicine, Karolinska Institutet, Solna, Stockholm, Sweden; 13 Regional Center for Health Care Improvement, St. Olav’s Hospital, Trondheim, Norway; 14 Center for Oral Health Services and Research (TkMidt), Trondheim, Norway

**Keywords:** Medications, Drugs, Pulmonary embolism, Drug discovery, Cardiology

## Abstract

Scandinavian electronic health-care registers provide a unique setting to investigate potential unidentified side effects of drugs. We analysed the association between prescription drugs dispensed in Norway and Sweden and the short-term risk of developing pulmonary embolism. A total of 12,104 pulmonary embolism cases were identified from patient- and cause-of-death registries in Norway (2004–2014) and 36,088 in Sweden (2005–2014). A case-crossover design was used to compare individual drugs dispensed 1–30 days before the date of pulmonary embolism diagnosis with dispensation in a 61–90 day time-window, while controlling for the receipt of other drugs. A BOLASSO approach was used to select drugs that were associated with short-term risk of pulmonary embolism. Thirty-eight drugs were associated with pulmonary embolism in the combined analysis of the Norwegian and Swedish data. Drugs associated with increased risk of pulmonary embolism included certain proton-pump inhibitors, antibiotics, antithrombotics, vasodilators, furosemide, anti-varicose medications, corticosteroids, immunostimulants (pegfilgrastim), opioids, analgesics, anxiolytics, antidepressants, antiprotozoals, and drugs for cough and colds. Mineral supplements, hydrochlorothiazide and potassium-sparing agents, beta-blockers, angiotensin 2 receptor blockers, statins, and methotrexate were associated with lower risk. Most associations persisted, and several additional drugs were associated, with pulmonary embolism when using a longer time window of 90 days instead of 30 days. These results provide exploratory, pharmacopeia-wide evidence of medications that may increase or decrease the risk of pulmonary embolism. Some of these findings were expected based on the drugs' indications, while others are novel and require further study as potentially modifiable precipitants of pulmonary embolism.

## Introduction

Pulmonary embolism (PE) arises when blood clots, often originating in deep veins, obstruct pulmonary arteries^1^. The development of PE encompasses an intricate interplay of pathophysiological, inflammatory, and chemical processes. These processes involve factors such as stasis, endothelial injury, hypercoagulability, inflammatory responses, damage to the endothelium, insults to the coagulation cascade (involving fibrin, thrombin, and tissue factor) as well as additional factors such as immobility and genetic predispositions^1^. The incidence of pulmonary embolism has increased globally over the last decades, particularly in high-income countries, while pulmonary embolism-related in-hospital death rate and age-standardized mortality from pulmonary embolism have decreased or plateaued^2–5^. Improved survival in patients with conditions predisposing to pulmonary embolism, such as cancer, chronic obstructive pulmonary disease, and autoimmune diseases, may contribute to the increased incidence rates, while a greater proportion of low-risk cases being diagnosed and improvements in the management of pulmonary embolism may have contributed to the reduced mortality rates ^2^. Several risk factors have been identified for pulmonary embolism, including age^6^, sex^6^, smoking^7^, obesity^8,9^, hypertension^7^, and physical inactivity^10^. In addition, surgery^11^, hospitalization, or trauma that leads to extended immobilization^12^, cancer^12,13^, pregnancy^14^, kidney disease^15^, heart failure^12^, or previous history of deep venous thromboembolism or pulmonary embolism are established risk factors. Some medications including oral contraceptives^16^, hormone replacement therapy^17^, tamoxifen^18–20^, bisphosphonates^21^, coagulants^22^, antidepressants^23^, and antipsychotics^24^ have been associated with increased risk, while statins have been associated with reduced risk^25^.

In the US, approximately 90% of adults aged 65 years and older use at least one prescription medication^26^. Side effects from use of drugs are a major public health concern and have been estimated to cost around $30 billion in the US^27^. Randomized trials used to test drugs are costly and therefore generally just large enough to detect an effect on the primary outcome of interest, which may be a physiological effect (e.g., blood pressure) rather than a hard clinical outcome. Less common, but important side effects may therefore be missed in many trials. In addition, pre-approval trials have tended to include fewer women, especially in their reproductive age (although some improvement has been observed in later years^28^), patients with comorbidities, children, and older adults, which may limit the generalizability of the findings from such studies to the general population^29^. Thus, systematic monitoring of the pharmaceutical effects of all approved drugs in clinical practice could advance clinical care and public health.

Our research group has previously conducted systematic examinations of all potential associations between prescribed drugs and short-term risk of acute myocardial infarction^30^ and ischemic stroke^31^, and others have used this approach to identify drugs that may improve or impair COVID-19 prognosis^32,33^. In the current study, we extended this approach to examine the association between prescribed medications and short-term risk of pulmonary embolism using comprehensive nationwide data in two countries.

## Methods

### Study design

A case-crossover design was used and methods have been described in detail previously^30,31^. This design included all cases of pulmonary embolism and applied self-matching by comparing exposure before disease onset with disease-free time in the past as a comparison control. A major advantage of the case-crossover design is that person-specific characteristics that are stable over the typically short-time periods such studies are conducted over (e.g. age, sex, lifestyle, chronic conditions) do not confound the observed associations.

### Dispensed medications

We assessed the risk of pulmonary embolism associated with every drug dispensed to patients that had a first-time pulmonary embolism within the study period. Data on dispensed medications prior to the diagnosis or death were extracted from the nation-wide registries of dispensed drugs in both Norway and Sweden. The Norwegian Prescription Database was established on January 1st 2004, and all Norwegian pharmacies are required to supply information on drug prescriptions including type, dose and date of dispensation^34^. Sweden established a similar registry on July 1st 2005, the Swedish National Prescribed Drug Register^35^. National personal identifiers attached to these data were used to link the information on drug prescriptions to other health-related registers existing in these countries. The databases do not include information on drugs purchased over-the-counter or drugs administered to institutionalized patients in nursing homes or hospitals. In Norway, it was possible to exclude participants who, at the time of their pulmonary embolism, were institutionalized and for whom registration of dispensed medications was not available. In Sweden, this information was not available, and therefore we included only those patients to whom at least one drug was dispensed during the year preceding the occurrence of pulmonary embolism. In Sweden, prescriptions are in general valid for one year after date of being issued^36^, and for chronic medication are refilled every third month. A similar system is in place in Norway.

### Outcome assessment

The Norwegian Patient Register (established in 2008)^37^, the Swedish National Patient Register (established in 1964)^38^, and the cause of death registries in Norway (established in 1951)^39^ and Sweden (established in 1952)^40^ were used to identify cases of pulmonary embolism. A total of 12,104 pulmonary embolism cases were identified from patient- and cause-of-death registries in Norway (2004–2014) and 36,088 in Sweden (2005–2014). The quality of the information in the Norwegian and Swedish patient registers has been shown to be very high for other cardiovascular outcomes^38,41,42^, and acceptable for acute pulmonary embolism in Sweden^43^. In Norway, all patients registered with either a primary hospital discharge diagnosis or underlying cause of death of ICD-10 I26 from 1 January 2008 to 31 December 2014 were included, and in Sweden, the corresponding dates for hospital diagnosis and cause of death were between 1 November 2005 and 31 December 2014. Only the first registered episode of pulmonary embolism was included in the analysis for each participant.

### Statistical analysis

For each patient, the occurrence of drug dispensing within 1–30 days before the date of pulmonary embolism occurrence (case period) was compared to a time window of 61–90 days before pulmonary embolism diagnosis (control period) for each drug individually. A 30 day wash-out period between the case- and control-periods was used to minimize the carryover effects of drugs. We calculated odds ratios together with 95% confidence intervals comparing the odds of drug dispensed in the case period to that in the control period using conditional logistic regression. The statistical models were adjusted for every other drug. Adjustments for other drugs were made at the fifth ATC level—so each drug investigated was adjusted for every other drug investigated. Additional analyses were conducted to examine the robustness of the findings where we extended the case-, control- and wash-out periods from 30 to 90 days (case period = 1–90 days before event occurrence, control period 181–270 days before event occurrence) and repeated all analyses. The 30 days^44^ and 90 days^44–46^ exposure periods were based on prior publications^44–46^.

We assessed the association between all dispensed medications and pulmonary embolism risk. Because we aimed to estimate the most likely effect size for drugs with true associations while accounting for simultaneous prescriptions, we did not use Bonferroni correction or similar conventional methods to address the issue of multiple comparisons, as they may fail to estimate the size of these associations correctly^47^. Instead, we applied a version of the least absolute shrinkage and selection operator (LASSO) regression analysis^47–51^ called bootstrap-enhanced least absolute shrinkage operator (BOLASSO)^52^. Several bootstrap samples are drawn from the dataset, where each bootstrap sample is generated by sampling N pairs (N is the total number of drugs in the dataset) with replacement. We drew 1000 bootstrap samples in this analysis. Of note, confidence intervals generated via the BOLASSO approach are not optimal, because each bootstrap sample is estimated on different penalty parameters. We include confidence intervals for ease of interpretation. As a result, drugs selected by this approach may include one (i.e., the null) within the confidence intervals. In BOLASSO, we obtain multivariable-adjusted estimates as the effect of each selected drug is controlled for the effects of all other selected drugs. In Supplementary Material, Online Appendix A, we present in detail the background of the method and how we implemented BOLASSO in conditional logistic regression models for case-crossover data.

Separate analyses were conducted for the Norwegian and Swedish data and we present both country-specific and combined estimates, using meta-analysis to combine the country-specific estimates. Drugs selected by BOLASSO with risk estimates in the same direction for both countries were considered a common hit and were included in the final analysis. A fixed-effects model was used to calculate the combined estimate^53^. All statistical analyses were conducted using R (version 3.2.3; R foundation for Statistical Computing, Vienna, Austria) and Stata/IC 16 (Stata Corp, College Station, Texas, USA).

### Ethical approval

The studies were approved by the Regional Committee for Medical and Health Research Ethics (REC) in Central Norway and Regional Ethical Review Board in Sweden. In addition, Norwegian data was also approved by the Norwegian Data Protection Authority. All methods were performed in accordance with the relevant guidelines and regulations by the respective ethical committees from both Norway and Sweden. Exemption from the requirement of obtaining informed consent from the registered individuals was given by REC. In all data files the personal identifier was replaced with a study allocation number as part of the data preparation by the data providers.

## Results

A total of 48,192 pulmonary embolism cases were included across the two countries, 36,088 from Sweden (33,678 identified via the patient register and 2410 via the cause of death register) and 12,104 from Norway (11,947 and 157 identified via the corresponding Norwegian registers). Characteristics of these individuals are presented in Table 1.

**Table 1:** Characteristics of the study sample.

Characteristics	Total N (%)	Sweden N (%)	Norway N (%)
Total PE patients*	48,192	36,088	12,104
Death due to PE	2567 (5.32)	2410 (6.68)	157 (1.30)
Males	22,563 (46.82)	16,743 (46.39)	5910 (48.83)
Age (in categories)			
30–39	1345 (2.79)	860 (2.38)	485 (4.01)
40–49	2694 (5.59)	1820 (5.04)	874 (7.22)
50–59	4731 (9.82)	3286 (9.11)	1445 (11.94)
60–69	10,316 (21.41)	7,697 (21.33)	2619 (21.64)
70–79	13,124 (27.23)	10,114 (28.03)	3010 (24.87)
80–89	12,906 (26.78)	9924 (27.50)	2982 (24.64)
90–99	3044 (6.32)	2364 (6.55)	680 (5.62)
> 100	32 (0.06)	23 (0.06)	9 (0.07)

*The number reflects the patients who were either hospitalized or died due to pulmonary embolism (PE) (ICD10-I26). In addition, the number (proportion) reflect the patients who dispensed prescribed medication either in the case-period (1–30 days) or control-period (61–90 days) before the index date for the diagnosis of PE in Norway (2008–2014) and Sweden (2004–2014), respectively.

**Figure 1:**
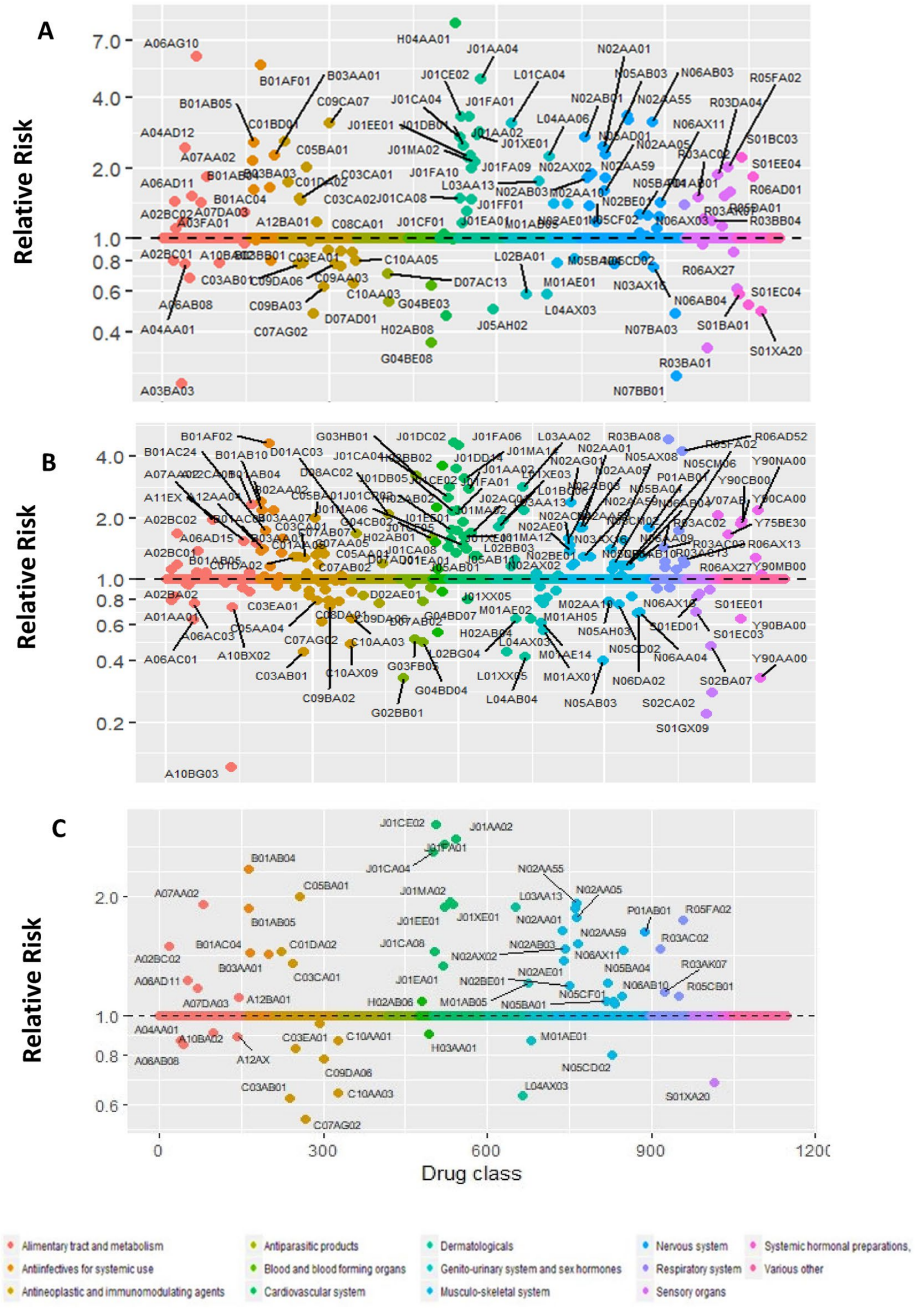
Case-crossover analysis of dispensed prescription medication use and risk of pulmonary embolism. The above plot illustrates (**A**) unique drug types which were selected in Norway, (**B**) unique drug types which were selected in Sweden, and (**C**) 59 drugs which were common hits from both the countries. Y-axis displays relative risk on the log scale, X-axis displays all the prescribed drugs studied grouped by the anatomical therapeutic chemical (ATC) classification.

A total of 1100 and 1260 distinct pharmaceutical drugs were dispensed among patients who experienced a subsequent pulmonary embolism in Norway and Sweden, respectively. Out of these, 773 unique drugs were dispensed in either the case- or control-period, in Norway and 1091 in Sweden. Of these, BOLASSO selected 117 unique drugs in Norway (Supplementary Table 1) and 191 in Sweden (Supplementary Table 2). Finally, a total of 59 drugs were selected in common from both countries (Fig. 1). Table 2 presents the country-specific and combined estimates of these dually-selected drugs.

**Table 2:** Relative risks (95% CIs) of pulmonary embolism within 30 days after the drug was dispensed, selected by BOLASSO approach in both countries.

Type of drug	ATC code	Drug names	Sweden	Norway	Total (Norway and Sweden pooled)
			RR (95% CI)	RR (95% CI)	RR (95% CI)
Proton pump inhibitors	A02BC02	Pantoprazole	1.44 (1.21–1.72)	1.68 (1.24–2.27)	1.50 (1.29–1.74)
Antiemetics and antinauseants	A04AA01	Ondansetron	0.78 (0.61–1.01)	0.94 (0.77–1.14)	0.87 (0.73–1.04)
Drugs for treating constipation	A06AB08	Sodium picosulfate	0.68 (0.47–0.98)	0.98 (0.85–1.11)	0.85 (0.60–1.21)
	A06AD11	Lactulose	1.52 (1.07–2.15)	1.08 (0.96–1.22)	1.23 (0.89–1.70)
Antibiotics	A07AA02	Nystatin	1.82 (1.18–2.80)	1.94 (1.55–2.44)	1.91 (1.57–2.34)
Antipropulsives	A07DA03	Loperamide	1.42 (0.95–2.10)	1.08 (0.89–1.31)	1.17 (0.92–1.49)
Anti-diabetic drug	A10BA02	Metformin	0.79 (0.59–1.06)	0.96 (0.82–1.11)	0.91 (0.76–1.08)
Mineral supplements	A12AX	Calcium, combinations with vitamin D and/or other drugs	0.95 (0.78–1.16)	0.87 (0.78–0.96)	0.89 (0.81–0.97)
	A12BA01	Potassium chloride	1.29 (0.88–1.89)	1.08 (0.92–1.28)	1.11 (0.95–1.29)
Antithrombotics	B01AB04	Dalteparin	2.15 (1.63–2.84)	2.40 (2.05–2.80)	2.34 (2.04–2.68)
	B01AB05	Enoxaparin	2.56 (1.86–3.53)	1.37 (1.05–1.80)	1.86 (1.01–3.43)
	B01AC04	Clopidogrel	1.61 (1.07–2.43)	1.40 (1.14–1.72)	1.44 (1.20–1.73)
Antianemic drug	B03AA01	Ferrous glycine sulfate	2.26 (0.75–6.79)	1.34 (0.88–2.03)	1.43 (0.97–2.12)
Vasodilators	C01DA02	Glyceryl trinitrate	1.73 (1.31–2.28)	1.28 (1.12–1.46)	1.45 (1.08–1.94)
Diuretics	C03AB01	Bendroflumethiazide and potassium	0.78 (0.52–1.16)	0.44 (0.23–0.85)	0.62 (0.36–1.08)
	C03CA01	Furosemide	1.48 (1.26–1.74)	1.27 (1.17–1.36)	1.35 (1.16–1.56)
	C03EA01	Hydrochlorothiazide and potassium-sparing agents	0.79 (0.53–1.17)	0.83 (0.72–0.97)	0.83 (0.72–0.95)
Anti-varicose drugs	C05BA01	Organo-heparinoid	2.02 (1.20–3.42)	2.00 (1.63–2.45)	2.00 (1.66–2.42)
Beta-blockers	C07AG02	Carvedilol	0.48 (0.29–0.79)	0.62 (0.39–0.99)	0.55 (0.39–0.78)
Angiotension II receptor blockers (ARBs)	C09CA06	Candesartan	0.89 (0.69–1.16)	0.98 (0.85–1.12)	0.96 (0.85–1.08)
	C09DA06	Candesartan and diuretics	0.78 (0.56–1.08)	0.78 (0.61–1.02)	0.78 (0.64–0.96)
Lipid-lowering agents	C10AA01	Simvastatin	0.87 (0.75–1.01)	0.87 (0.79–0.95)	0.87 (0.80–0.94)
	C10AA03	Pravastatin	0.64 (0.40–1.03)	0.64 (0.41–1.00)	0.64 (0.46–0.89)
Corticosteroids for systemic use	H02AB06	Prednisolone	1.04 (0.91–1.20)	1.12 (1.02–1.23)	1.09 (1.01–1.18)
Anti-thyroid drugs	H03AA01	Levothyroxine sodium	0.99 (0.82–1.20)	0.87 (0.79–0.97)	0.90 (0.81–1.02)
Antibiotics	J01AA02	Doxycycline	2.84 (2.32–3.47)	2.75 (2.47–3.07)	2.77 (2.52–3.05)
	J01CA04	Amoxicillin	2.69 (2.20–3.28)	2.51 (2.14–2.95)	2.58 (2.28–2.92)
	J01CA08	Pivmecillinam	1.48 (1.19–1.82)	1.43 (1.21–1.67)	1.45 (1.27–1.65)
	J01CE02	Phenoxymethylpenicillin	3.31 (2.81–3.89)	2.82 (2.51–3.16)	3.02 (2.59–3.53)
	J01EA01	Trimethoprim	1.30 (0.94–1.79)	1.35 (1.07–1.70)	1.33 (1.10–1.61)
	J01EE01	Sulfamethoxazole and trimethoprim	2.22 (1.50–3.29)	1.73 (1.34–2.23)	1.87 (1.49–2.35)
	J01FA01	Erythromycin	3.29 (2.41–4.49)	2.17 (1.52–3.09)	2.70 (1.79–4.05)
	J01MA02	Ciprofloxacin	2.11 (1.63–2.75)	1.90 (1.66–2.17)	1.94 (1.72–2.19)
	J01XE01	Nitrofurantoin	2.76 (1.74–4.39)	1.41 (1.14–1.73)	1.91 (0.99–3.67)
Antineoplastic and immunomodulating agents	L03AA13	Pegfilgrastim (immunostimulant)	1.76 (0.91–3.43)	2.18 (0.80–5.95)	1.88 (1.08–3.27)
	L04AX03	Methotrexate (immunosuppresant)	0.58 (0.35–0.98)	0.64 (0.48–0.84)	0.63 (0.49–0.80)
Anti-inflammatory and antirheumatics	M01AB05	Diclofenac	1.40 (1.18–1.65)	1.07 (0.97–1.19)	1.21 (0.93–1.58)
	M01AE01	Ibuprofen	0.79 (0.61–1.02)	0.92 (0.77–1.11)	0.87 (0.75–1.02)
Opioids	N02AA01	Morphine	2.45 (1.40–4.26)	1.78 (1.50–2.11)	1.86 (1.50–2.32)
	N02AA05	Oxycodone	1.59 (1.19–2.12)	1.80 (1.58–2.05)	1.76 (1.57–1.99)
	N02AA55	Oxycodone and naloxone	2.28 (1.04–5.01)	1.78 (1.07–2.96)	1.92 (1.25–2.94)
	N02AA59	Codeine, combinations excl. psycholeptics	1.80 (1.57–2.07)	1.29 (1.14–1.45)	1.52 (1.10–2.11)
	N02AB03	Fentanyl	1.81 (1.08–3.03)	1.59 (1.21–2.08)	1.64 (1.29–2.08)
	N02AE01	Buprenorphine	1.37 (0.90–2.08)	1.38 (1.06–1.80)	1.38 (1.10–1.72)
	N02AX02	Tramadol	1.88 (1.55–2.27)	1.16 (1.05–1.29)	1.47 (0.91–2.35)
Analgesics	N02BE01	Paracetamol	1.17 (1.02–1.33)	1.19 (1.13–1.27)	1.19 (1.13–1.25)
Anxiolytics	N05BA01	Diazepam	1.06 (0.86–1.30)	1.12 (0.93–1.34)	1.09 (0.95–1.25)
	N05BA04	Oxazepam	1.26 (1.02–1.55)	1.19 (1.06–1.33)	1.21 (1.09–1.33)
Hypnotics and sedatives	N05CD02	Nitrazepam	0.84 (0.58–1.20)	0.75 (0.50–1.10)	0.80 (0.61–1.04)
	N05CF01	Zopiclone	1.02 (0.91–1.16)	1.16 (1.06–1.28)	1.09 (0.97–1.24)
	N05CF02	Zolpidem	1.25 (0.88–1.78)	1.05 (0.94–1.18)	1.07 (0.96–1.19)
Antidepressants	N06AB10	Escitalopram	1.10 (0.87–1.39)	1.16 (0.88–1.54)	1.12 (0.94–1.35)
	N06AX11	Mirtazapine	1.42 (1.00–2.02)	1.47 (1.26–1.72)	1.46 (1.27–1.69)
Antiprotozoals	P01AB01	Metronidazole	1.39 (0.96–2.02)	1.78 (1.37–2.32)	1.63 (1.29–2.06)
Drugs for obstructive airway disease	R03AC02	Salbutamol	1.50 (1.21–1.87)	1.44 (1.19–1.74)	1.47 (1.27–1.69)
	R03AK07	Formoterol and budesonide	1.21 (0.90–1.62)	1.14 (1.00–1.31)	1.15 (1.02–1.30)
Drugs for cough and cold preparations	R05CB01	Acetylcysteine	1.12 (0.91–1.37)	1.12 (1.00–1.25)	1.12 (1.00–1.25)
	R05FA02	Opium derivatives and expectorants	2.01 (1.14–3.55)	1.73 (1.53–1.95)	1.74 (1.55–1.96)
Other opthalmologicals	S01XA20	Artificial tears and other indifferent preparations	0.49 (0.32–0.77)	0.89 (0.74–1.06)	0.68 (0.38–1.22)

*ATC code* anatomical therapeutic chemical (ATC), 5th level.

### Cardiovascular drugs

Several cardiovascular drugs were associated with higher risk of pulmonary embolism, some of which could be due to their indication of use. The antithrombotic agents dalteparin, enoxaparin, and clopidogrel were associated with increased risk, while the angiotensin 2 receptor blocker ‘candesartan and diuretics’ was associated with reduced risk. Diuretics yielded mixed results, with lower risk for hydrochlorothiazide and potassium-sparing agents, while furosemide was associated with higher risk. The vasodilator glyceryl trinitrate was associated with increased risk. An inverse association was observed for the beta-blocking agent carvedilol.

### Antibiotics

Several antibiotics examined were associated with increased risk. This includes nystatin, doxycycline, amoxicillin, pivmecillinam, phenoxymethylpenicillin, trimethoprim, sulfamethoxazole and trimethoprim combined, erythromycin, ciprofloxacin, and nitrofurantoin.

### Opioids

Several opioids were associated with higher pulmonary embolism risk, including morphine, oxycodone, oxycodone and naloxone combined, codeine combinations excluding psycholeptics, fentanyl, buprenorphine, and tramadol.

### Antineoplastic and immunomodulating agents

A positive association was observed for pegfilgrastim, while lower risk was observed for methotrexate.

### Other medications

Analgesics (paracetamol), antiprotozoals (metronidazole), anti-varicose therapy (organo-heparinoid), anxiolytic (oxazepam, but not diazepam), proton pump inhibitors (pantaprazole, but not omeprazole) and corticosteroids for systemic use (prednisolone), but not corticosteroids for dermatological use (clobetasol) were associated with increased risk of pulmonary embolism. Positive associations were observed for cough and cold preparations, such as acetylcysteine and opium derivatives and expectorants. An inverse association was observed for calcium supplements combined with vitamin D or other drugs/supplements.

### Additional analyses with a longer exposure window

In additional analyses, when we extended the case-, control and wash-out periods from 30 to 90 days, many of the results remained similar, while others were either stronger or weaker than in the primary analysis (Supplementary Table 3). Additional positive associations were observed for ACE inhibitors (enalapril, ramipril), adrenergics/drugs for obstructive airway diseases (salbutamol, terbutaline, indacaterol, formoterol and budenoside), angiotensin II receptor blockers (losartan), antianemics (ferrous sulfate), antibiotics (azithromycin), antidepressants (sertraline, mianserin), antiemetics and antinauseants (aprepitant), antifibrinolytics (tranexamic acid), anti-inflammatory and anti-rheumatic agents (etoricoxib), antimyotics (fluconazole), antineoplastic and immunomodulating agents (capecitabine, erlotinib, tamoxifen), antipropulsives (loperamide), antipsychotics (haloperidol, risperidone), anxiolytics (diazepam), cardiac glycosides (digoxin), corticosteroids for systemic use (dexamethasone), drugs for treating constipation (sodium picosulfate), estrogens (estriol), hormonal contraceptives (levonorgestrel and ethinylestradiol, drospirenone and ethinylestradiol), hypnotics (zopiclone, zolpidem), intravaginal ring (with progestogen and estrogen), opioids (tramadol), and proton pump inhibitors (esomeprazole) (Supplementary Table 3). In addition, the positive associations between prednisolone and pulmonary embolism was substantially strengthened (Supplementary Table 3). Additional inverse associations were observed between anti-inflammatory and anti-rheumatic agents (glucosamine), antithrombotics (warfarin), calcium channel blockers (felodipine), and drugs for urinary frequency and incontinence (tolterodine) and pulmonary embolism (Supplementary Table 3). Supplementary Tables 4, 5 shows the unique drugs selected from each country using the 90 day time window, but also includes hits that were not common.

## Discussion

This study systematically investigated the associations between all drugs requiring a prescription and short-term risk of developing pulmonary embolism. Using nationwide registry data from Norway and Sweden, and employing an exposure-wide approach, we identified 38 drugs that were either associated with higher (31 drugs) or lower (seven drugs) short-term risk of pulmonary embolism. In additional analyses, using a longer exposure period, which may be more appropriate for some drugs, 66 drugs were associated with higher risk and seven with lower risk.

Antibiotics tended to be most consistently associated with higher risk of pulmonary embolism, and we observed higher risk across several types of antibiotics examined. However, these associations are likely to be confounded by underlying infection, which is a strong risk factor for pulmonary embolism^11,54,55^, as it might lead to increased inflammation and triggering of platelets, resulting in fibrin deposition and thrombus formation^56^. We also cannot exclude the possibility of reverse causation, where premonitory symptoms of pulmonary embolism like dyspnea are empirically presumed to reflect the more common occurrence of respiratory infection.

Prescription of various opioids was also quite consistently associated with higher risk of pulmonary embolism. This may reflect an increased risk of pulmonary embolism during and after surgery^13,57^. Almost 25% of all cases of venous thromboembolism occur during or shortly after surgery^12^. Similarly, the positive associations observed for certain antithrombotics also likely reflects confounding by indication, as these drugs are used to reduce the risk of pulmonary embolism during or after surgery.

The positive association between the antidepressant mirtazapine and pulmonary embolism is consistent with the Million Women’s Study, which reported a positive association between antidepressant use and risk of venous thromboembolism with pulmonary embolism^23^. However, a case-control study reported no association^58^.

We observed a positive association between drugs used to treat cough and cold (e.g., acetylcysteine, and opium derivatives and expectorants) and obstructive airway disease (e.g., salbutamol, formoterol and budesonide) and risk of pulmonary embolism. An acute infection or exacerbation could potentially explain these associations.

Blood pressure-lowering drugs had differing associations with pulmonary embolism risk. While inverse associations were observed for carvedilol, hydrochlorothiazide, and for candesartan in combination with diuretics, furosemide was associated with increased risk. Some large cohort studies have suggested an inverse association between blood pressure and pulmonary embolism risk^59,60^. It is possible that the increased pulmonary embolism risk with use of furosemide could be due to activation of the renin–angiotensin–aldosterone system^61^, which has a pro-thrombotic effect^62^. This, on the other hand, could explain why angiotensin-2 receptor blockers reduce the risk of pulmonary embolism, especially in a possible subpopulation that requires a combination of angiotensin-2 receptor blockers and diuretics and may have higher risk of atherosclerotic disease^63^. Beta blockers, and mainly unselected beta blockers, such as carvedilol, can reduce increased sympathetic activity with their prothrombotic effect and thereby reduce pulmonary embolism risk^64^. However, other potential explanations may be reverse causation, whereby premonitory dyspnea is presumed to reflect congestive heart failure, confounding by indication, perhaps by kidney disease^65^, or acute dehydration by furosemide and increased risk of pulmonary embolism as part of the classical Virchow’s triad^66^.

The inverse association observed between dispensed statins (simvastatin and pravastatin) and pulmonary embolism is consistent with results from a cohort study^25^ showing reduced incidence from pulmonary embolism with use of statins. The results are also partly consistent with meta-analyses of randomized controlled trials, showing reduced incidence from venous thromboembolism with use of statins^67,68^. However, in these meta-analyses, only one trial specifically focused on pulmonary embolism and reported an imprecise effect estimate (HR = 0.77, 0.41–1.46). The association between serum cholesterol level with risk of pulmonary embolism is not clear^6,69–71^, and thus it is possible that the observed benefit with statins is due to other non-specific effects unrelated to lipid-lowering, such as anti-inflammatory effects^72^.

There was a weak inverse association observed between calcium supplementation combined with vitamin D or other drugs and risk of pulmonary embolism (HR = 0.89, 0.81–0.97). The Women’s Health Initiative randomized controlled trial reported a similar, but less precise, association between calcium and vitamin D supplementation in relation to pulmonary embolism risk (HR = 0.92, 95% CI 0.73–1.16)^73^.

The strength of this study includes the large-scale registry-based databases from Norway and Sweden and the comprehensive assessment of a wide range of drugs in relation to short-term risk of pulmonary embolism. These registers are of high quality and near complete. Case-crossover studies are vulnerable to misclassification^74^, however, in our study, we could minimize some of the sources of bias due to misclassification. Both countries have a tax-financed universal healthcare system that is accessible to all residents and reporting to the health registers is obligatory. Recall bias with regard to the exposure was eliminated by relying on recorded data. Moreover, the outcome, i.e. pulmonary embolism, can be identified with high degree of accuracy due to the modern diagnostic methods and the universal healthcare in Scandinavia^43^, reducing the potential for selection bias. Use of nationwide databases also supports the generalisability of the findings. The large sample size provided sufficient statistical power to detect even modest associations.

Several of the observed findings likely reflect confounding by indication, and we were not able to separate the effect of a drug and its indication in the current study. It is therefore unclear whether the observed associations were due to the drugs themselves or due to the underlying or prodromal conditions the drugs were meant to treat. However, this type of confounding may be less likely to explain the results when observing inverse associations or when different medications within the same group of medications are differentially associated with pulmonary embolism. In such cases, a direct effect of a drug on pulmonary embolism is more likely. Another limitation of the study is that we did not have detailed information on lifestyle-related risk factors or comorbidities that potentially may have acted as effect modifiers. Most previous studies on drugs and pulmonary embolism were observational cohort and case-control studies^23,25,58,75^. We did not identify prior case-crossover studies on medications and pulmonary embolism risk. Thus, our comparison with previous literature is also limited by differences in design and potential latency periods between drug prescription and the development of pulmonary embolism. The current case-crossover design is best suitable to identify acute or triggering effects of drugs on outcomes with a sudden onset, but may misestimate the effects of long-term drug use on risk(76). It was beyond this project to assess potential drug interactions, but this may be a point for further investigation in future studies. The databases did not include information on over-the-counter drugs or drug prescriptions in hospitals or nursing homes, however, in Norway institutionalized subjects without registration of dispensed medications were excluded, while in Sweden only subjects with at least one drug dispensed in the year preceding the occurrence of pulmonary embolism were included. Lastly, the prescription databases only contain information on the date of dispensing, and not information on the date of actual administration of drugs or whether the drug was taken, but this likely resulted in non-differential misclassification and bias toward the null.

In conclusion, we found both positive and inverse associations between a wide range of dispensed prescription drugs and short-term risk of pulmonary embolism. Further studies are needed to explore these findings in more detail. This method presents an opportunity for systematic and thorough monitoring of a wide range of drugs that could trigger or prevent the onset of serious health conditions, including pulmonary embolism.

### Supplementary Information

Supplementary Information 1.

Supplementary Information 2.

Supplementary Tables.

## Data Availability

The data used in the current analysis are available from the Norwegian Patient Register, Norwegian Prescription Database, Norwegian Cause of Death Registry, and Swedish National Patient Register, Swedish National Prescribed Drug Register, Swedish National Cause of Death Register, but restrictions apply to the availability of these data, which were used under license for the current study, and are therefore not publicly available. Requests for data access should be addressed to Abhijit Sen. Email: abhijit.sen@ntnu.no.
